# An Error in the Lippe Repertory

**Published:** 1892-01

**Authors:** S. Mills Fowler

**Affiliations:** Gainesville, Texas


Editor of The Homœopathic Physician:

### AN ERROR IN THE LIPPE REPERTORY.

Gainesville, Texas, Dec. 6th, 1891.

Editor of The Homœopathic Physician:

Some time since I discovered something wrong with my copy of Lippe's *Repertory*, and of course it is the same in every other copy of the edition.

In chapter xxvi, "chest and heart," pp. 191 and 192. At the bottom of p. 191 is paragraph, "heart, palpitation of," ending with "*Oleand.*" Then turn over to p. 192, at the bottom is paragraph, "heaviness (weight), sensation in chest," ending with "Ox-ac." Neither of these paragraphs is complete on the same page upon which it was begun. Now look at the top of p. 191 and there will be found the ending of both paragraphs.

I have give the two paragraphs some study and comparison, and have decided that the continuation of the paragraph on p. 191 is at the third line from top of p. 192, while the completion of the paragraph on the bottom of p. 192 is the true line at the top of the same page.

Will you please call the attention of the profession to the error, and if I am not right, let us try and find out what is.

Yours truly,

S. Mills Fowler.

